# Meta-analysis and trial sequential analysis of shexiang baoxin pill for coronary slow flow

**DOI:** 10.3389/fphar.2022.955146

**Published:** 2022-08-22

**Authors:** Hongxin Guo, Xingyuan Li, Mingjun Zhu

**Affiliations:** ^1^ First Clinical Medical College, Henan University of Chinese Medicine, Zhengzhou, China; ^2^ Department of Cardiovascular Diseases, The First Affiliated Hospital of Henan University of Chinese Medicine, Zhengzhou, China

**Keywords:** Shexiang Baoxin Pill, coronary slow flow, Meta-analysis, Grade, trial sequential analysis

## Abstract

**Background:** Coronary slow flow (CSF) is a common cardiovascular phenomenon with no effective treatment in conventional Western medicine (CWM). Shexiang Baoxin Pill (SXBXP) is a widely used Chinese medicine for cardiovascular disease in China, and clinical studies have shown that it has good efficacy for CSF.

**Objective:** To systematically evaluate the efficacy and safety of SXBXP for CSF.

**Methods:** Seven databases were searched to identify related randomized controlled trials (RCTs). The Meta-analysis, trial sequential analysis (TSA), and Grades of Recommendation, Assessment, Development and Evaluation (GRADE) were performed using Stata 14.1, TSA 0.9.5.10 Beta and GRADE profiler 3.2.2 software respectively.

**Results:** A total of 10 RCTs were included. Meta-analysis showed that compared with CWM treatment alone, SXBXP combined with CWM further improved the angina pectoris efficacy [RR = 1.37, 95% CI (1.23, 1.52), *p* < 0.000 01] and nitric oxide (NO) level [WMD = 11.32, 95% CI (0.04, 22.59), *p* = 0.049], decreased the mean corrected TIMI frame count (CTFC) [WMD = −4.23, 95% CI (−5.51, −2.95), *p* < 0.000 01], CTFC of the left anterior descending artery (LAD) [WMD = −6.36, 95% CI (−12.07, −0.65), *p* = 0.029], left circumflex artery (LCX) [WMD = -5.73, 95% CI (−8.79, −2.67), *p* < 0.000 01], and right coronary artery (RCA) [WMD = −6.72, 95% CI (−10.60, −2.84), *p* = 0.001], decreased the positive rate of treadmill exercise test [RR = 0.45, 95% CI (0.25, 0.83), *p* = 0.010], endothelin-1 (ET-1) level [WMD = -11.03, 95% CI (−13.92, −8.14), *p* < 0.000 01], high-sensitivity C-reactive protein (hs-CRP) [WMD = −1.95, 95% CI (−2.57, −1.34), *p* < 0.000 01], and adverse reactions [RR = 0.20, 95% CI (0.05, 0.85), *p* = 0.030]. The GRADE evidence quality rating presented with moderate, low or very low quality of evidence. TSA further affirmed the clinical efficacy.

**Conclusion:** Although some results suggest that there may be a positive effect of SXBXP for CSF, the quality of the primary study including the reporting is too poor and therefore, no benefits could be demonstrated. More high-quality studies are still needed to further confirm the efficacy and safety.

**Systematic review registration:** https://www.crd.york.ac.uk/PROSPERO/, identifier (CRD42022329469).

## 1 Introduction

Coronary slow flow (CSF) is a cardiovascular disease with angina pectoris as the main symptom, except for cardiomyopathy, connective tissue disease, structural heart disease, coronary artery dilation and spasm, and no significant stenosis on coronary angiography, but with delayed perfusion in the distal coronary arteries, the disease was first proposed by Tambe et al. (1972) (Chalikias and Tziakas, 2021). CSF is not a rare disease, and its diagnostic tools are relatively single, with an incidence of approximately 1%–7% in patients undergoing diagnostic angiography (Wang and Nie, 2011; Montone et al., 2020). As a chronic disease of non-instrumental lesions, CSF patients are easily seen due to recurrent chest pain attacks, which seriously reduce their quality of life (Sun et al., 2021). However, the pathogenesis of CSF has not been fully elucidated by modern medicine, and there are no large clinical studies on the treatment of the disease, resulting in no effective uniform treatment protocol for the time being (Sun et al., 2021). Therefore, it is important to explore the methods of traditional Chinese medicine (TCM) with exact effect and easy to use.

According to the relevant clinical manifestations of CSF, the disease belongs to the categories of “chest painful impediment” and “heart pain” in TCM (Mou and Rong, 2020). In recent years, more and more scholars have tried to use TCM for CSF and confirmed its effectiveness, which has led to an increasing interest in the role of TCM in improving CSF. In 2021, Li et al. (2021) reviewed the pathogenesis of CSF and the treatment strategy of TCM. They finally concluded that the core pathogenesis of CSF is blood stasis, and the combination of Chinese and Western medicine can achieve better results. In the same year, Huang and Feng focused on reviewing the progress of combined Chinese and Western medicine treatment of CSF and found that a variety of Chinese herbal medicines, Chinese patent medicines, and Chinese herbal injections have better efficacy on it, among which Shexiang Baoxin Pill (SXBXP) is the most used (Huang and Feng, 2021). SXBXP is currently one of the most commonly used aromatic medicines for the treatment of cardiovascular diseases in China, which has been widely used to relieve and prevent angina-related symptoms since its marketing in 1981 (Ge et al., 2020). It is composed of Moschus Artifactus (Shexiang), Styrax (Suhexiang), Ginseng Radix Et Rhizoma (Renshen), artificial Bovis Calculus (Niuhuang), Cinnamomi Cortex (Rougui), Bufonis Venenum (Chansu), and Borneolum Syntheticum (Bingpian). The details of SXBXP are summarized in Supplementary Material S1, including the source, composition and extraction procedure, as well as its actions, indications, administration and so on. In recent years, a number of clinical studies on SXBXP in the treatment of CSF have been carried out, but the findings are controversial about their effectiveness and safety, and most of them are single-center, small-sample studies with uneven study quality, which have not been systematically evaluated. It can be seen that although increasing studies have shown that SXBXP may have an effect on improving CSF, the existing evidence still fails to draw reliable conclusions, which limits its popularization to a certain extent. Therefore, this study systematically and rigorously screened the literature that met the criteria and used meta-analysis to conduct a comprehensive analysis of the clinical efficacy and safety of SXBXP in the treatment of CSF. In addition, unlike most meta-analyses, this study will use trial sequential analysis (TSA) for sample size estimation to minimize false-positive results due to small sample size or random error. The Grades of Recommendation, Assessment, Development and Evaluation (GRADE) is a globally harmonized system for grading the quality of evidence and the strength of recommendations, mainly for meta-analysis evaluation and clinical guideline development (Guyatt et al., 2008). This study will also evaluate the quality of evidence for the outcomes using the GRADE system to provide more comprehensive evidence to better guide clinical practice.

## 2 Information and methods

The Preferred Reporting Items for Systematic Reviews and Meta-Analyses (PRISMA) criteria were followed while conducting this Meta-analysis (Supplementary Material S2), which was registered in the International Prospective Register of Systematic Reviews (number: CRD42022329469). The abbreviations used in this manuscript are listed in Supplementary Material S3.

### 2.1 Inclusion and exclusion criteria

Inclusion criteria were as follows 1) Study type: Randomized controlled trials (RCTs) of SXBXP for the treatment of CSF in the Chinese and English languages. 2) Patients: Patients who meet the diagnostic criteria for CSF, regardless of disease duration, gender, age, and region. The diagnostic criteria were referred to the corrected TIMI frame count (CTFC) method of Gibson et al. (1996) and CSF was diagnosed when CTFC was >27 frames after coronary angiography. 3) Intervention measures: The experimental group and control group were both treated with antiplatelet drugs, lipid-lowering drugs, vasodilators of nitrate and other conventional western medicine (CWM). The experimental group was added with SXBXP (produced by Shanghai Hutchison Pharmaceuticals Co., Ltd.) for adjuvant therapy. 4) Outcomes: 1) Efficacy on angina pectoris (assessment criteria refer to Guiding principles for clinical research of new Chinese medicines) (Zheng, 2002), overall response rate = (markedly effective + effective)/total number of cases × 100%; 2) CTFC; 3) Positive rate of treadmill exercise test (determination criteria refer to Guiding principles for clinical research of new Chinese medicines) (Zheng, 2002); 4) Endothelin-1 (ET-1) level; 5) Nitric oxide (NO) level; 6) High-sensitivity C-reactive protein (hs-CRP) level; 7) Adverse reactions.

Exclusion criteria were as follows 1) Patients with coronary heart disease, heart failure, cardiomyopathy, connective tissue disease, structural heart disease, coronary artery dilatation and spasm and other influencing factors; 2) Repeated literatures; 3) Obvious errors in study data; 4) Incomplete date; 5) The baseline information of patients was inconsistent; 6) Outcomes were not consistent with those specified in this study; 7) Case reports, conference reports, famous doctors’ experience, animal experiments, reviews, and similar literatures; 8) Intervention measures combined with other TCM.

### 2.2 Literature search strategy and data extraction

Two investigators (Guo HX and Li XY) independently searched the China National Knowledge Infrastructure (CNKI), Wanfang Data Knowledge Service Platform (Wanfang), VIP, China Biology Medicine disc, PubMed, Cochrane Library, and Embase databases from inception until May 2022. The method for searching was based on subject headings combined with free words, the terms searched included “coronary”, “coronary artery”, “slow flow”, “coronary slow flow”, “Shexiang Baoxin Pill”, and “Randomized controlled trial”. Take PubMed as an example. The detailed search strategy is shown in Supplementary Material S4.

Two investigators independently (Guo HX and Li XY) performed literature screening and full-text reading, and independently extracted the data of the finally included literatures. The contents extracted mainly include basic information and general characteristics of the included studies, information related to literature quality evaluation, interventions, course of treatment, and outcomes, etc.

### 2.3 Literature quality evaluation and GRADE evidence quality rating

Two investigators (Guo HX and Li XY) evaluated the methodological quality of the included literatures according to the RCT risk of bias assessment tool in the Cochrane Handbook for Systematic Reviews, from seven aspects: random sequence, allocation concealment, blind method of patients and staff, blind method of outcome evaluators, integrity of outcome data, selective reporting of results, and other sources of bias, and each was rated as “low risk,” “unclear,” or “high risk” according to the results (Higgins et al., 2011).

The GRADE profiler 3.2.2 evidence quality grading system was used to rank the results of systematic reviews from five aspects: risk of bias, inconsistency, indirectness, imprecision, and publication bias of the studies, which were divided into four levels: high quality evidence, medium quality evidence, low quality evidence, and very low quality evidence (Guyatt et al., 2008).

### 2.4 Statistical analysis

In this study, STATA 14.1 software was used for meta-analysis. Binary variables were analyzed using relative risk (RR) as the pooled statistic, and continuous variables were analyzed using weighted mean difference (WMD) as the pooled statistic, both of which describe the 95% confidence interval (CI). Heterogeneity size was evaluated by *p* value and *I* ^*2*^ value. If the inter-study statistical heterogeneity was small (*I* ^*2*^ ≤ 50% or *p* ≥ 0.1), the fixed-effect model was used; if the inter-study heterogeneity was significant (*I* ^*2*^ > 50% or *p* < 0.1), the random-effect model was used, and the source of heterogeneity was explored by subgroup or sensitivity analysis. STATA 14.1 software was used for Begg’s and Egger’s tests for publication bias analysis, and if there was publication bias, the trim and fill method was used to evaluate whether publication bias influenced the results. *p* < 0.05 was considered statistically significant for the pooled effect. Finally, the TSA 0.9.5.10 Beta software was used to perform TSA analysis on the associated results.

## 3 Results

### 3.1 Literature search results and basic characteristics of the included literatures

**FIGURE 1:**
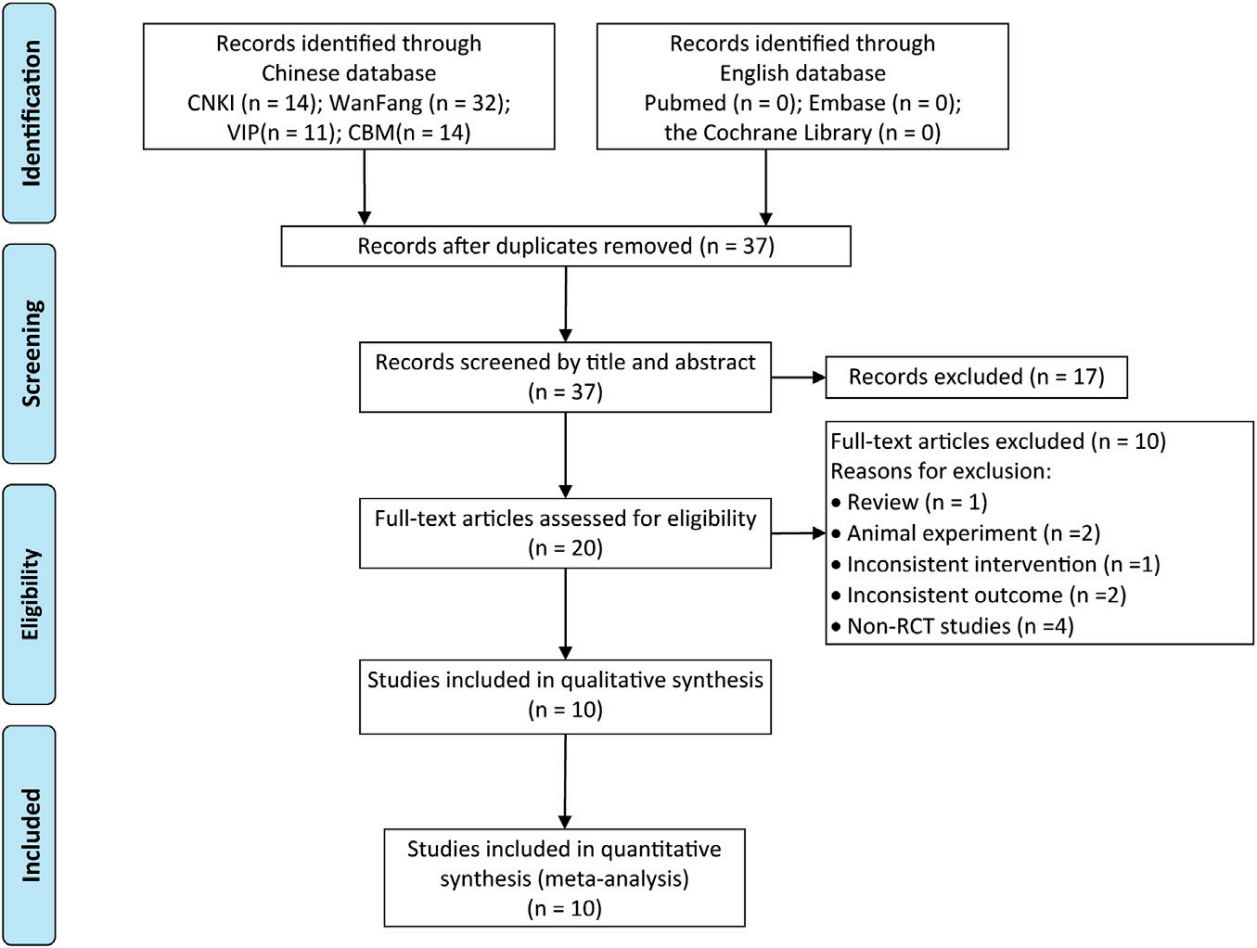
Literature screening process.

A total of 71 relevant literatures were obtained in the initial screening, 34 repeated literatures were excluded after checking, 17 literatures significantly inconsistent with the subject were excluded after preliminary reading of the title and abstract, the remaining 20 papers were read in full and 10 were excluded, including reviews, discrepancies in outcome indicators, retrospective studies, self-control studies, experimental animal studies, and discrepancies in interventions, and 10 RCTs were finally included (Chu et al., 2011; Wang and Tan, 2015; Zhou, 2015; Zhang et al., 2016; Yang and Zhou, 2017; Li et al., 2018; Feng, 2019; Wu et al., 2019; Huo, 2020; Shen et al., 2021). A total of 588 patients were included, with a course of treatment of 1 month (Yang and Zhou, 2017) for up to 12 months (Shen et al., 2021). The literature search results are shown in Figure 1; the basic characteristics of the included literatures are shown in Table 1.

**TABLE 1:** Basic information of the included studies.

Study	Age		Sample size		Gender (male/female)		Interventions		Duration (month)	Outcomes
	T	C	T	C	T	C	T	C		
Chu et al. (2011)	60.9 ± 15.1	57.7 ± 17.8	15	15	8/7	9/6	Shexiang Baoxin Pill + CWM	CWM	3	①
Feng (2019)	52.13 ± 4.55	52.03 ± 4.23	30	30	19/11	18/12	Shexiang Baoxin Pill + CWM	CWM	2	①②
Huo (2020)	51.78 ± 5.04	50.44 ± 5.56	38	38	27/11	26/12	Shexiang Baoxin Pill + CWM	CWM	3	①④⑤⑥
Li et al. (2018)	56.6 ± 6.4		30	30	19/11	17/13	Shexiang Baoxin Pill + CWM	CWM	6	①③
Shen et al. (2021)	41.38 ± 9.43	45.75 ± 10.61	32	32	20/12	18/14	Shexiang Baoxin Pill + CWM	CWM	12	②
Wang and Tan (2015)	55.4 ± 7.2		34	32	35/31		Shexiang Baoxin Pill + CWM	CWM	6	②④⑥
Wu et al. (2019)	53.7 ± 2.6	54.1 ± 2.5	38	38	24/14	23/15	Shexiang Baoxin Pill + CWM	CWM	3	①③④⑤⑥⑦
Yang and Zhou (2017)	58.3 ± 3.8	56.5 ± 3.6	22	23	10/12	13/10	Shexiang Baoxin Pill + CWM	CWM	1	①②③⑦
Zhang et al. (2016)	50.7 ± 6.3	51.3 ± 7.5	35	30	19/16	17/13	Shexiang Baoxin Pill + CWM	CWM	3	①④⑤⑥
Zhou (2015)	55.4 ± 7.2		24	22	25/21		Shexiang Baoxin Pill + CWM	CWM	6	②

**Note:** T, the experimental group; C, the control group; CWM, conventional western medicine, including antiplatelet drugs, statins lipid-lowering drugs, nicorandil, nitrates, etc.; Outcomes: ① Efficacy on angina pectoris, ② CTFC, ③ Positive rate of treadmill exercise test, ④ Endothelin-1 (ET-1), ⑤ Nitric oxide (NO), ⑥ High-sensitivity C-reactive protein (hs-CRP), ⑦ Adverse reactions.

### 3.2 Quality evaluation results of the included literatures

**FIGURE 2:**
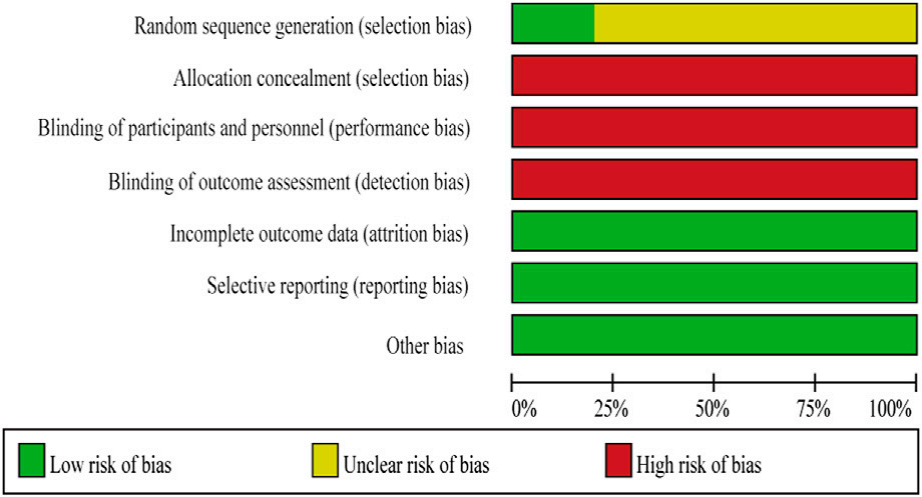
Risk of bias graph.

The 10 studies included in this study were all RCT studies, of which two studies (Zhang et al., 2016; Shen et al., 2021) described the correct mode of randomization in detail, and the remaining eight studies (Chu et al., 2011; Wang and Tan, 2015; Zhou, 2015; Yang and Zhou, 2017; Li et al., 2018; Feng, 2019; Wu et al., 2019; Huo, 2020) did not describe the mode of randomization; all studies did not report whether allocation concealment and blindness were performed; the data of all studies were complete; all studies specifically described the interventions and outcome measures; see Figure 2 and Supplementary Material S5

### 3.3 Results of meta-analysis, subgroup analysis and sensitivity analysis

#### 3.3.1 Efficacy on angina pectoris

**FIGURE 3:**
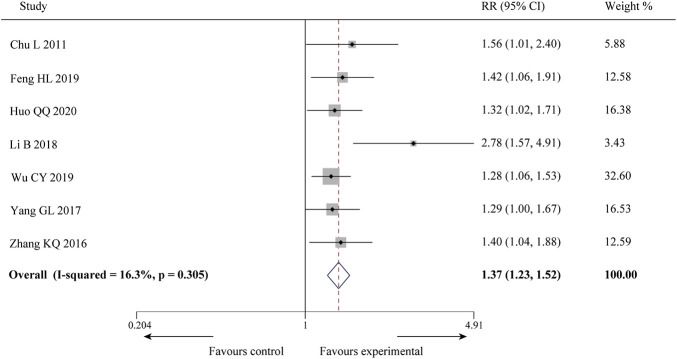
Forest plot of meta-analysis of efficacy on angina pectoris between the two groups.

Seven of the 10 studies (Chu et al., 2011; Zhang et al., 2016; Yang and Zhou, 2017; Li et al., 2018; Feng, 2019; Wu et al., 2019; Huo, 2020) reported the efficacy on angina pectoris, involving a total of 412 patients. There was little statistical heterogeneity among the studies (*p* = 0.305, *I* ^*2*^ = 16.3%), and a fixed-effect model was used for meta-analysis. The result showed that compared with CWM, the combination of SXBXP could effectively improve the angina pectoris efficiency [RR = 1.37, 95% CI (1.23, 1.52), *p* < 0.000 01], as detailed in Figure 3.

#### 3.3.2 Corrected TIMI frame count

**FIGURE 4:**
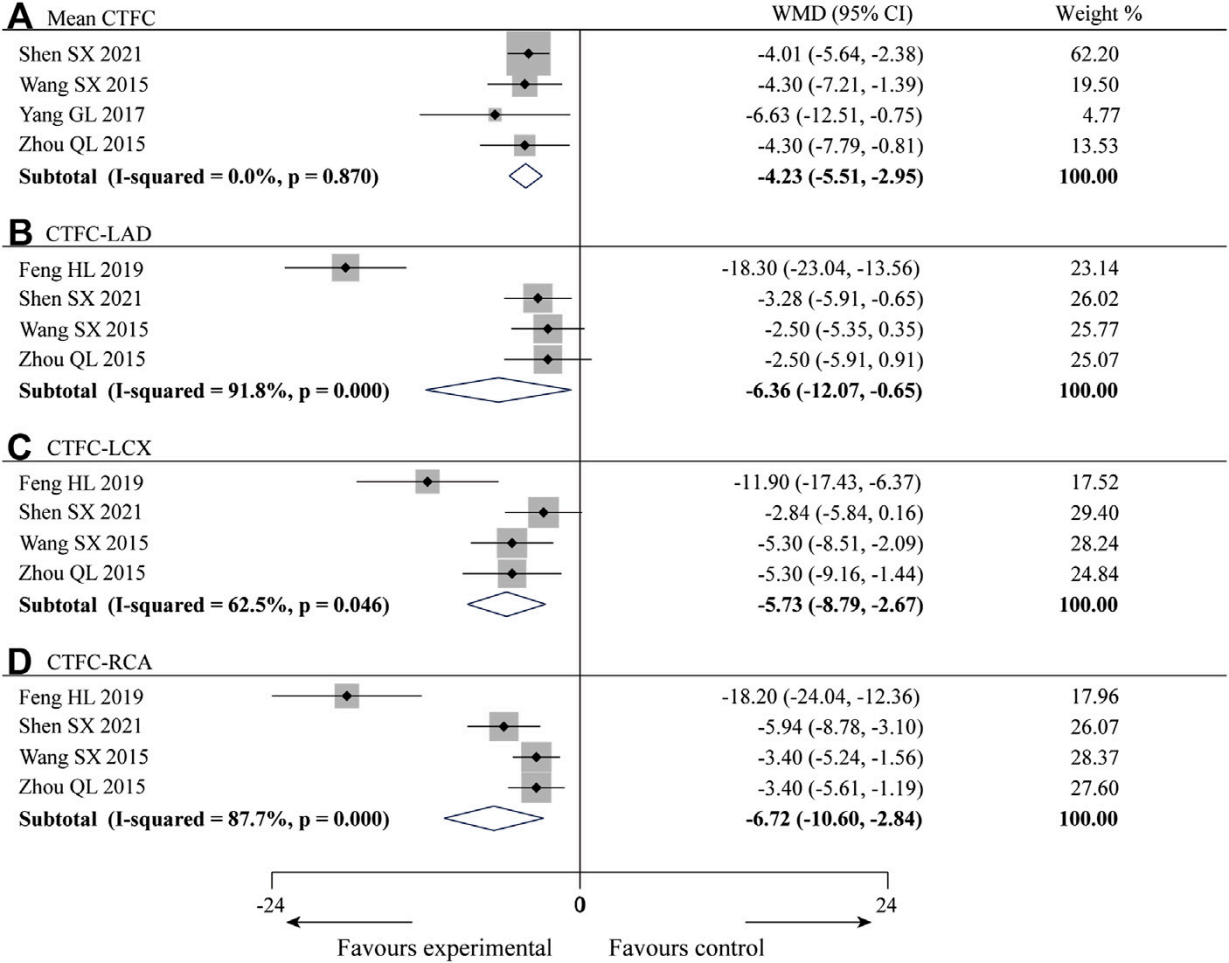
Forest plot of meta-analysis of mean CTFC **(A)**, CTFC-LAD **(B)**, CTFC-LCX **(C)** and CTFC-RCA **(D)** between the two groups.

Four of the 10 studies (Wang and Tan, 2015; Zhou, 2015; Yang and Zhou, 2017; Shen et al., 2021) reported the mean CTFC with a total of 221 patients. There was no statistical heterogeneity among the studies (*p* = 0.87, *I* ^*2*^ = 0%), and a fixed-effect model was used for meta-analysis. The results showed that compared with CWM, the combination of SXBXP could effectively reduce the mean value of CTFC [WMD = −4.23, 95% CI (−5.51, −2.95), *p* < 0.000 01], see Figure 4A for details*.* Four studies (Wang and Tan, 2015; Zhou, 2015; Feng, 2019; Shen et al., 2021) reported left anterior descending artery CTFC (CTFC-LAD), left circumflex artery CTFC (CTFC-LCX), and right coronary artery CTFC (CTFC-RCA), containing a total of 236 patients. The heterogeneity test results showed that the statistical heterogeneity of CTFC-LAD (*p* = 0.000, *I* ^*2*^ = 91.8%), CTFC-LCX (*p* = 0.046, *I* ^*2*^ = 62.5%) and CTFC-RCA (*p* = 0.000, *I* ^*2*^ = 87.7%) was high (Figures 4B–D), so subgroup analysis was performed to explore the sources of heterogeneity based on treatment duration (less than or more than 5 months), average age (less than or more than 55 years old), gender distribution (male account for less than or more than 60%) and sample size (less than or more than 62 patients). The results showed the heterogeneity was significantly reduced in subgroups of treatment duration more than 5 months, average age more than 55 years old, male account for less than 60% and sample size more than 62 patients (Table 2) (Supplementary Materials S6–S8), indicating that all these factors may be sources of heterogeneity. However, it is noteworthy that heterogeneity is still high in some of the remaining subgroups. Since CTFC testing requires coronary angiography by the interventionalist, the procedure is influenced by a variety of factors, resulting in a certain amount of error in the measurements from one hospital to another, and we consider that heterogeneity might result from this. In addition to the factors mentioned above, we found no other significant differences in clinical and methodological features among the four studies. We believed that these clinical differences are acceptable when exploring the efficacy of SXBXP in the whole population, so the random-effect model was used for meta-analysis. The results showed that compared with CWM, the combination with SXBXP could effectively reduce the CTFC of LAD [WMD = −6.36, 95% CI (−12.07, −0.65), *p* = 0.029], LCX [WMD = −5.73, 95% CI (−8.79, −2.67), *p* < 0.000 01] and RCA [WMD = −6.72, 95% CI (−10.60, −2.84), *p* = 0.001], see Figures 4B–D.

**TABLE 2:** Subgroup analysis of CTFC-LAD, CTFC-LCX, and CTFC-RCA based on treatment duration, average age, gender distribution, and sample size.

Outcomes	Criteria for grouping	Subgroup	n	WMD (95%CI)	*I* ^*2*^	*p*
CTFC-LAD	treatment duration	less than 5 months	1	−18.30 (−23.04, −13.56)	—	<0.000 01
		more than 5 months	3	−2.82 (−4.50, −1.14)	0.0%	<0.005
	average age	less than 55 years old	2	−10.66 (−25.37, 4.06)	96.6%	= 0.156
		more than 55 years old	2	−2.50 (−4.69, −0.31)	0.0%	<0.05
	gender distribution	male account for less than 60%	3	−2.82 (−4.50, −1.14)	0.0%	<0.005
		male account for more than 60%	1	−18.30 (−23.04, −13.56)	—	<0.000 01
	sample size	less than 62 patients	2	−10.31 (−25.79, 5.17)	96.4%	= 0.192
		more than 62 patients	2	−2.92 (−4.85, −0.99)	0.0%	<0.005
CTFC-LCX	treatment duration	less than 5 months	1	−11.90 (−17.43, −6.37)	—	<0.000 01
		more than 5 months	3	−4.31 (−6.22, −2.40)	0.0%	<0.000 01
	average age	less than 55 years old	2	−7.06 (−15.92, 1.80)	87.4%	= 0.118
		more than 55 years old	2	−5.30 (−7.77, −2.83)	0.0%	<0.000 01
	gender distribution	male account for less than 60%	3	−4.31 (−6.22, −2.40)	0.0%	<0.000 01
		male account for more than 60%	1	−11.90 (−17.43, −6.37)	—	<0.000 01
	sample size	less than 62 patients	2	−8.29 (−14.73, −1.85)	72.8%	<0.05
		more than 62 patients	2	−4.00 (−6.41, −1.59)	16.8%	<0.005
CTFC-RCA	treatment duration	less than 5 months	1	−18.20 (−24.04, −12.36)	—	<0.000 01
		more than 5 months	3	−3.90 (−5.17, −2.64)	18.9%	<0.000 01
	average age	less than 55 years old	2	−11.79 (−23.80, 0.21)	92.7%	= 0.054
		more than 55 years old	2	−3.40 (−4.81, −1.99)	0.0%	<0.000 01
	gender distribution	male account for less than 60%	3	−3.90 (−5.17, −2.64)	18.9%	<0.000 01
		male account for more than 60%	1	−18.20 (−24.04, −12.36)	—	<0.000 01
	sample size	less than 62 patients	2	−10.54 (−25.04, 3.95)	95.4%	= 0.154
		more than 62 patients	2	−4.43 (−6.87, −1.99)	53.9%	<0.000 01

**Note:** n, number of studies; CTFC, corrected TIMI, frame count; LAD, left anterior descending artery; LCX, left circumflex artery; RCA, right coronary artery.

#### 3.3.3 Positive rate of treadmill exercise test

**FIGURE 5:**
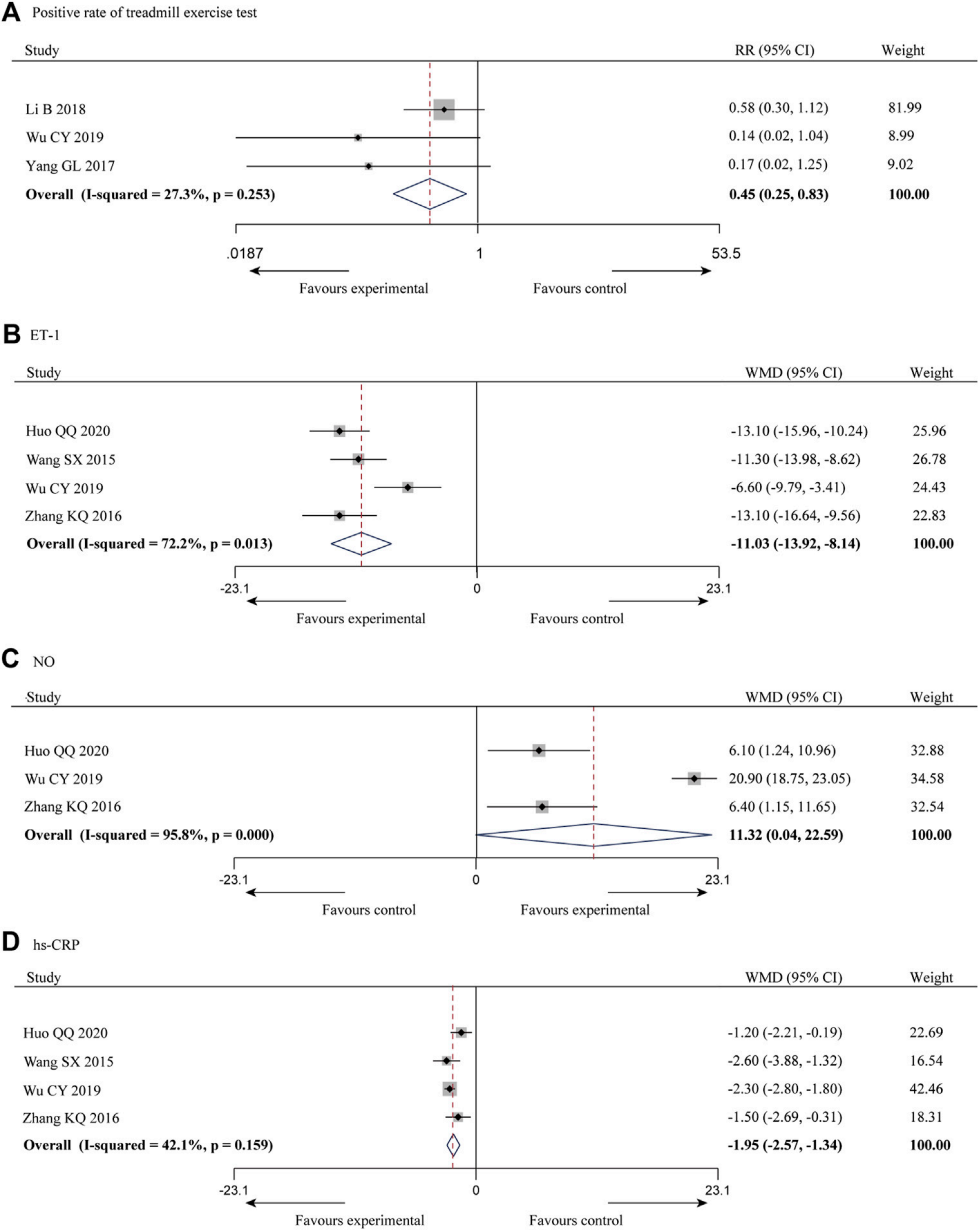
Forest plot of meta-analysis of the positive rate of treadmill exercise test **(A)**, ET-1 **(B)**, NO **(C)** and hs-CRP **(D)** between the two groups.

Three of the 10 studies (Li et al., 2018; Wu et al., 2019; Yang and Zhou, 2017) reported the positive rate of treadmill exercise test, with a total of 165 patients and less heterogeneity among the studies (*p* = 0.253, *I* ^*2*^ = 27.3%), and a meta-analysis was performed using a fixed-effect model, and the results showed that the combination of SXBXP could effectively reduce the incidence of positive treadmill exercise test compared with CWM [RR = 0.45, 95% CI (0.25, 0.83), *p* = 0.010], as detailed in Figure 5A.

#### 3.3.4 Endothelin-1

ET-1 is a powerful vasoconstrictor peptide that can contract coronary arteries to increase blood flow resistance, which is closely related to multiple coronary disease (Schiffrin, 2001; Ruaro et al., 2019). It was reported in four of the 10 studies (Wang and Tan, 2015; Zhang et al., 2016; Wu et al., 2019; Huo, 2020), with a total of 283 patients, and the inter-study heterogeneity was high (*p* = 0.013, *I* ^*2*^ = 72.2%) (Figure 5B). Subgroup analysis was also conducted based on the above criteria to explore the source of heterogeneity. Since all four studies had a sample size of more than 62 patients, we did not conduct subgroup analysis based on sample size. The results showed the heterogeneity was significantly reduced in subgroups of male account for less than 60% (Table 3) (Supplementary Material S9), indicating that gender distribution may be a source of heterogeneity. In addition, considerable heterogeneity remained in the remaining subgroups (Table 3) (Supplementary Material S9), suggesting that heterogeneity may come from other sources. Same reason as CTFC, random-effect model was used for meta-analysis. The results showed that the combination of SXBXP could effectively reduce ET-1 compared with CWM [WMD = −11.03, 95% CI (−13.92, −8.14), *p* < 0.000 01], as shown in Figure 5B.

**TABLE 3:** Subgroup analysis of ET-1 and NO based on treatment duration, average age and gender distribution.

Outcomes	Criteria for grouping	Subgroup	N	WMD (95%CI)	*I* ^*2*^	*p*
ET-1	treatment duration	less than 5 months	3	−10.93 (−15.19, −6.67)	81.4%	<0.000 01
		more than 5 months	1	−11.30 (−13.98, −8.62)	—	<0.000 01
	average age	less than 55 years old	3	−10.93 (−15.19, −6.67)	81.4%	<0.000 01
		more than 55 years old	1	−11.30 (−13.98, −8.62)	—	<0.000 01
	gender distribution	male account for less than 60%	2	−11.95 (−14.09, −9.82)	0.0%	<0.000 01
		male account for more than 60%	2	−9.89 (−16.26, −3.52)	88.7%	<0.005
NO	gender distribution	male account for less than 60%	1	6.40 (1.15, 11.65)	—	<0.05
		male account for more than 60%	2	13.67 (−0.83, 28.17)	96.6%	= 0.065

**Note:** n, number of studies; ET-1, endothelin-1; NO, nitric oxide.

#### 3.3.5 Nitric oxid

NO was reported in three of the 10 studies (Zhang et al., 2016; Wu et al., 2019; Huo, 2020), including 217 patients, with great inter-study heterogeneity (*p* = 0.000, *I* ^*2*^ = 95.8%) (Figure 5C). Subgroup analysis was also conducted based on the above criteria to explore the source of heterogeneity. Since the treatment duration, average age and sample size of the three studies were relatively consistent, we could only conduct subgroup analysis by gender distribution. But the result showed that gender distribution was not the cause of heterogeneity (Table 3) (Supplementary Material S10), sensitivity analysis was therefore performed to further find the cause of heterogeneity. After the study of Wu et al., 2019 was removed, the heterogeneity was significantly decreased (*I* ^*2*^ = 0.0%). Further studies found that in the intervention measures of Wu et al., 2019, nicorandil tablet was used in both experimental and control groups, while nicorandil was not used in the other studies, which may be the reason for heterogeneity. The results of random-effect model pooled analysis showed that the combination with SXBXW could effectively increase the NO level compared with CWM [WMD = 11.32, 95% CI (0.04, 22.59), *p* = 0.049], as detailed in Figure 5C.

#### 3.3.6 Hs-CRP

Hs-CRP was reported in four of the 10 studies (Wang and Tan, 2015; Zhang et al., 2016; Wu et al., 2019; Huo, 2020), involving a total of 283 patients, with less heterogeneity among the studies (*p* = 0.159, *I* ^*2*^ = 42.1%). The results of fixed effect model pooled analysis showed that compared with CWM, the combination of SXBXP could effectively reduce the hs-CRP level [WMD = -1.95, 95% CI (−2.57, −1.34), *p* < 0.000 01], as shown in Figure 5D *.*

#### 3.3.7 Adverse reactions

Adverse reactions were reported in two of the 10 studies (Yang and Zhou, 2017; Wu et al., 2019), with a total of 121 patients, of which one study (Yang and Zhou, 2017) reported no adverse reactions during the trial, so it could not be pooled; the results of one study (Wu et al., 2019) showed that two patients in the experimental group had adverse reactions and 10 patients in the control group had adverse reactions, with a total number of 38 patients in both groups. The meta-analysis results showed that the incidence rate of adverse reactions in the experimental group was lower than that in the control group [RR = 0.20, 95% CI (0.05, 0.85), *p* = 0.030].

### 3.4 Publication bias

**FIGURE 6:**
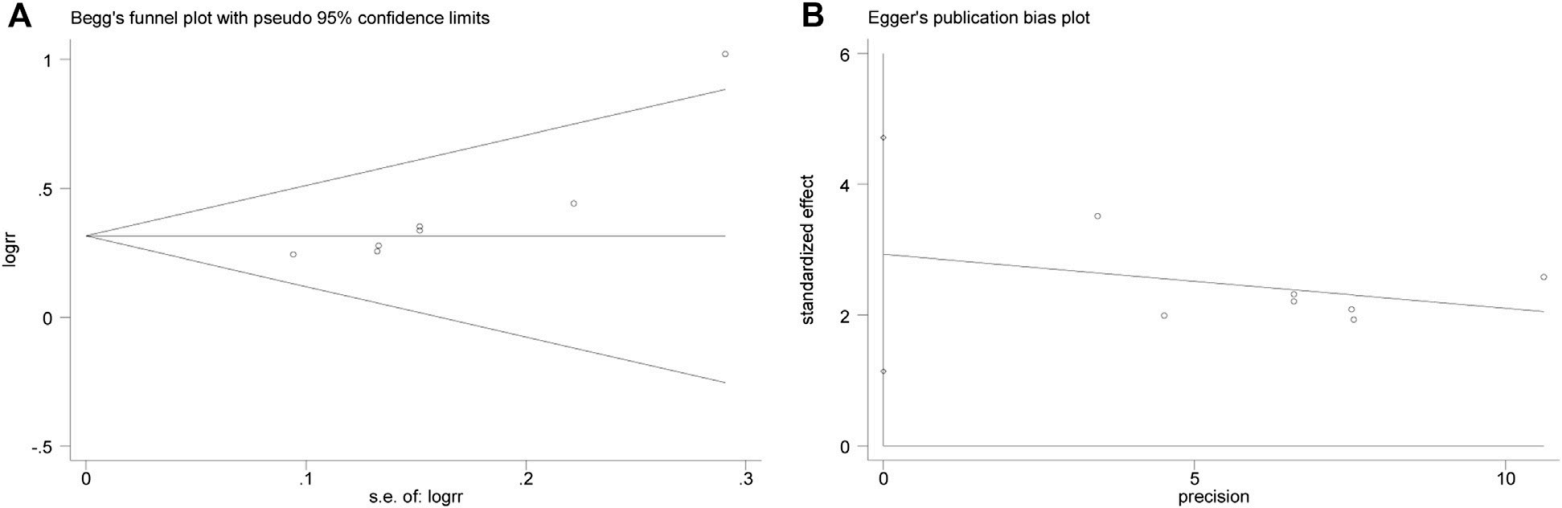
Begg **(A)** and Egger **(B)** test for efficacy on angina pectoris.

Begg (Figure 6A) and Egger (Figure 6B) tests were performed for the efficacy on angina pectoris, resulting in *t* = 4.20, *p* = 0.008, indicating the possibility of publication bias in the efficacy on angina pectoris of the outcome measures.

**FIGURE 7:**
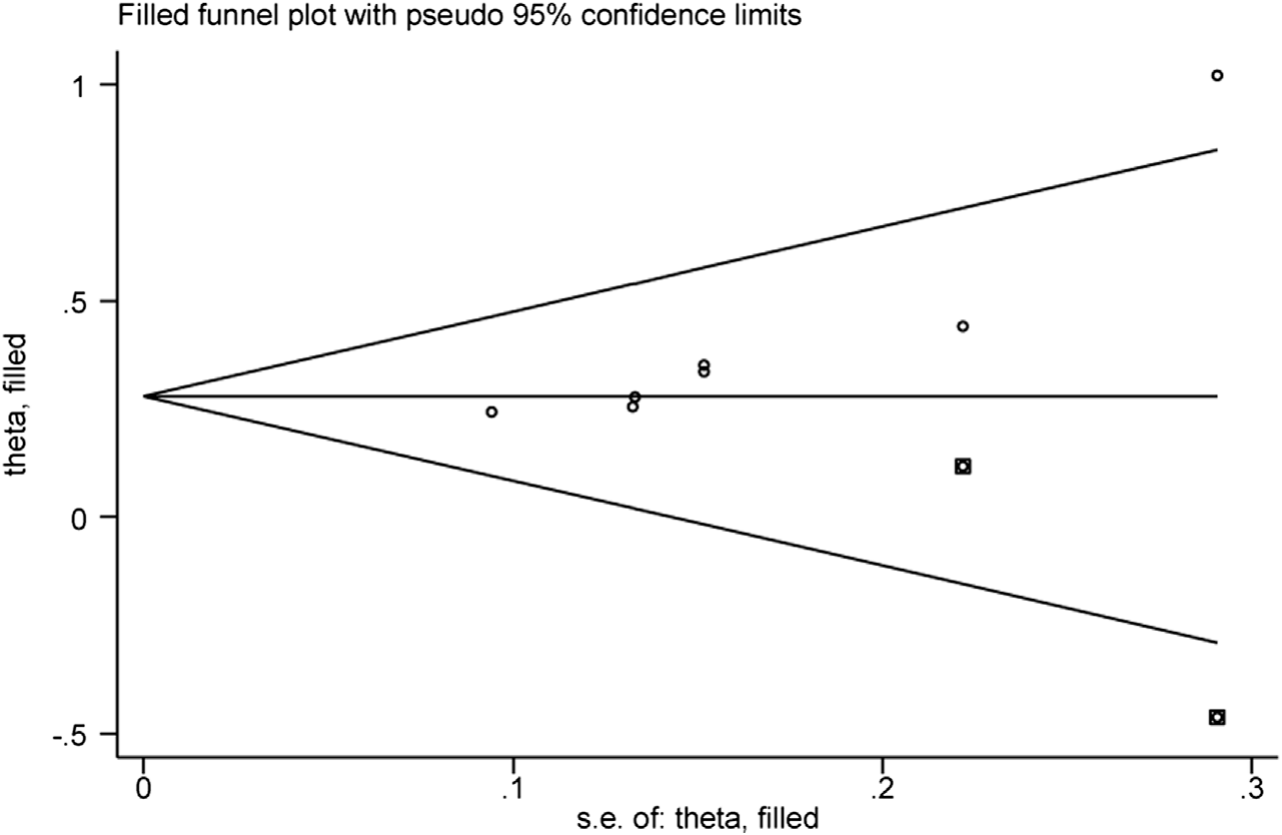
Funnel plot of the trim and fill method.

Then the effect of publication bias on the final results was evaluated by the trim and fill method. The fixed effect model logRR before trim-and-fill was 0.32, and its 95% CI was (0.209, 0.420). After iteration by trim-and-fill, the logRR changed to 0.28, and its 95% CI changed to (0.179, 0.381). The 95% CI was statistically significant before and after trim-and-fill. After trim-and-fill and supplement of two points to eliminate the effect of publication bias, the center of gravity of the funnel plot did not change significantly, indicating that the publication bias had no significant effect on the pooled results, and the results were robust and credible. See Figure 7 for details, and the square points in Figure 7 are supplementary points.

### 3.5 GRADE evidence quality rating

The results of the meta-analysis were evaluated for the GRADE quality evidence, in which mean CTFC and hs-CRP were moderate quality evidence, the efficacy on angina pectoris, CTFC-LAD, CTFC-LCX, positive rate of treadmill exercise test, ET-1, NO, and adverse reactions were low quality evidence, and CTFC-RCA was very low quality evidence (Table 4).

**TABLE 4:** GRADE evidence evaluation.

Outcomes	Number of studies	Risk of bias	Inconsistency	Indirectness	Imprecision	Publication bias	Grade of quality evidence
Efficacy on Angina Pectoris	7	Serious^h^	None	None	None	Presence^f^	Low
Mean CTFC	4	Serious^h^	None	None	None	None	Moderate
CTFC-LAD	4	Serious^h^	Serious^a^	None	None	None	Low
CTFC-LCX	4	Serious^h^	Serious^b^	None	None	None	Low
CTFC-RCA	4	Serious^h^	Major^c^	None	None	Presence^g^	Very Low
Positive Rate of Treadmill Exercise Test	3	Serious^h^	None	None	Serious^i^	None	Low
ET-1	4	Serious^h^	Serious^d^	None	None	None	Low
NO	3	Serious^h^	Serious^e^	None	None	None	Low
Hs-CRP	4	Serious^h^	None	None	None	None	Moderate
Adverse Reactions	2	Serious^h^	Serious	None	Serious^i^	None	Low

Note: CTFC, corrected TIMI, frame count; LAD, left anterior descending artery; LCX, left circumflex artery; RCA, right coronary artery; ET-1, endothelin-1; NO, nitric oxide; hs-CRP, high-sensitivity C-reactive protein.

a I^2^ = 91.8%.

b I^2^ = 62.5%

c I^2^ = 87.7%.

d I^2^ = 72.2%.

e I^2^ = 95.8%.

f For Begg and Egger’s test *p* = 0.008.

g For Begg and Egger’s test *p* = 0.021.

h Missing of blindness, with inadequate allocation concealment.

i The number of studies and sample size are small.

### 3.6 Trial sequential analysis

**FIGURE 8:**
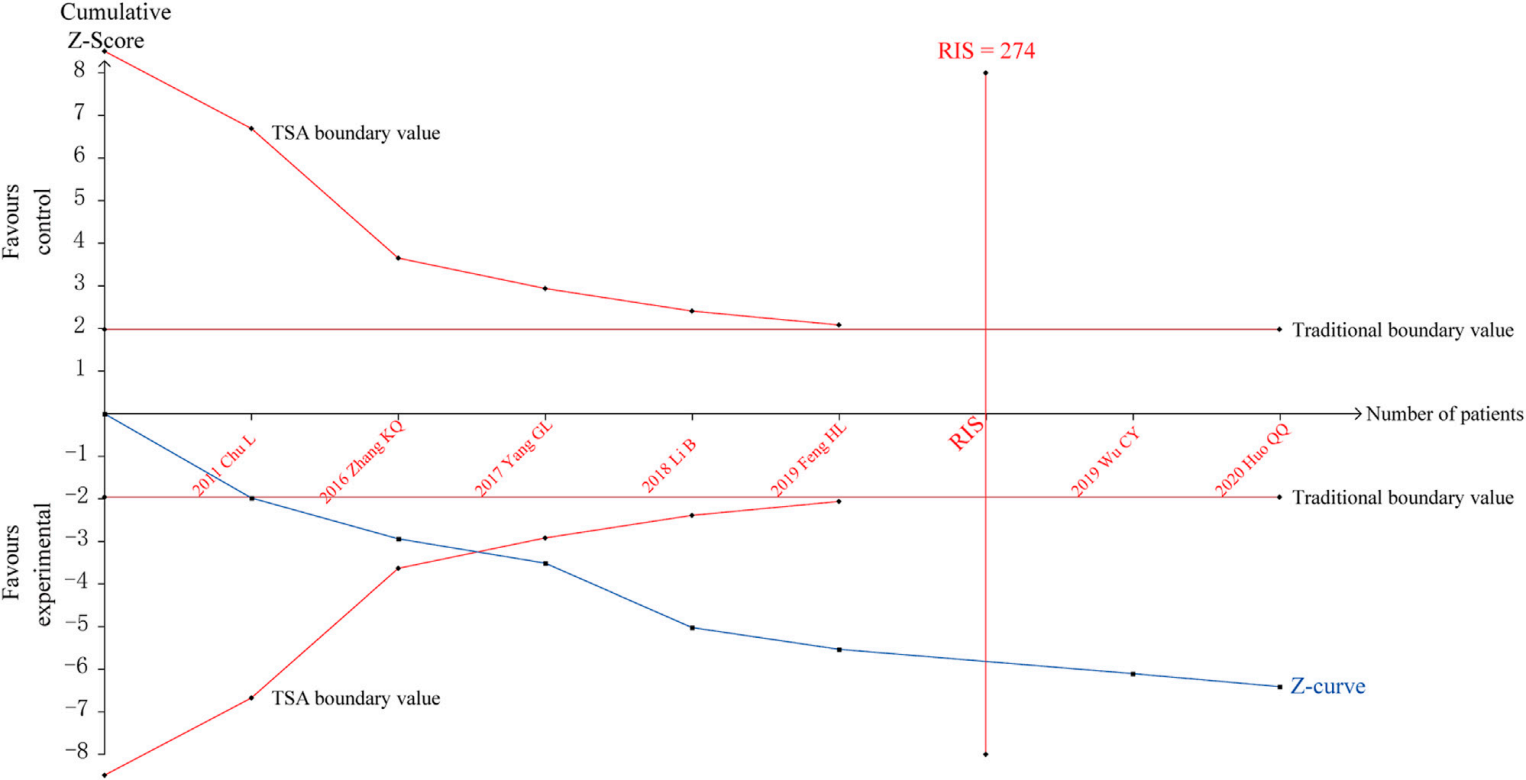
Trial sequential analysis for the efficacy on angina pectoris.

TSA was performed for the efficacy on angina pectoris. The parameters of this study were set as follows: type I error probability *α* = 5%, statistical power 1-β = 80%, and RR reduced by 20%. The sample size was used as the required information size (RIS) for two-sided test. The results showed that the cumulative *Z* value crossed the traditional boundary value and TSA boundary values after the third study was included, a positive conclusion was reached in advance, and the RIS curve was crossed after the sixth study, indicating that the sample size of the study had reached the expected value, as shown in Figure 8.

A penalty test was performed on the TSA results, setting the probability of type I error *α* = 5%, the penalty value *λ* as 2, and the type of bounds as a two-sided test. The penalized Z-curve exceeded the traditional boundary value of *Z* = 1.96, further affirming the effectiveness of SXBXP in treating CSF, as shown in Supplementary Material S11.

## 4 Discussion

We included a total of 10 RCT studies in this study and systematically evaluated the efficacy and safety of SXBXP in the treatment of CSF and observed its effects on the efficacy on angina pectoris, CTFC, positive rate of treadmill exercise test, ET-1, NO, hs-CRP and adverse reactions. The results showed that SXBXP combined with CWM was superior to CWM alone in improving the efficacy on angina pectoris and NO level, reducing mean CTFC, CTFC-LAD, CTFC-LCX, CTFC-RCA, reducing the positive rate of treadmill exercise test, ET-1 level, hs-CRP level, and incidence of adverse reactions. It means that SXBXP can improve angina related symptoms, accelerate coronary blood flow, improve vascular endothelial function, and reduce inflammatory factors. In terms of safety, the available results indicate that SXBXP may be safe. However, only two studies reported adverse drug reactions, which were insufficient to provide convincing evidence. TSA further affirmed the efficacy of SXBXP. In addition, the results of GRADE evidence quality rating showed that mean CTFC and hs-CRP were of moderate quality, angina pectoris efficacy, CTFC-LAD, CTFC-LCX, positive rate of treadmill exercise test, ET-1, NO, and adverse reactions were of low quality, and CTFC-RCA was of very low quality. The GRADE rating results show that these results are not reliable.

The pathogenesis of CSF still is not clear, and it is mostly considered to be related to coronary atherosclerosis, endothelial dysfunction, chronic inflammatory response, oxidative stress response, microvascular dysfunction, and abnormal platelet function (Li et al., 2019; Henein and Vancheri, 2021; Zhu and Su, 2021). SXBXP is a modified formula of Su He Xiang Pill derived from Taiping Huimin Heji Ju Fang in the Song Dynasty and it is indicated for the chest painful impediment syndrome with pattern of Qi stagnation and blood stasis. It was initially mainly used to relieve the immediate symptoms of angina pectoris attacks, and it is now mostly used to prevent angina pectoris attacks (Huangfu et al., 2017). In the formula, Moschus Artifactus (Shexiang) has the functions of promoting blood circulation, removing blood stasis, and opening the orifices to relieve pain, and is a sovereign drug; Styrax (Suhexiang) has the effect of aromatic herbs activating Yang, Ginseng Radix Et Rhizoma (Renshen) can benefit Qi and activate stagnancy, and both of them are minister drugs; artificial Bovis Calculus (Niuhuang) can open the orifices and induce resuscitation, Cinnamomi Cortex (Rougui) can warm Yang to promote coronary circulation, and Bufonis Venenum (Chansu) can open the orifices to relieve pain; Borneolum Syntheticum (Bingpian) can open the orifices to clear heat and relieve pain, and it is an envoy drug, together they exert aromatic and warming effects, benefitting Qi and strengthening the heart (Fan et al., 2018).

In this study, the efficacy on angina pectoris and the positive rate of treadmill exercise test mainly reflect the improvement of angina pectoris symptoms and exercise tolerance, which is closely related to the quality of life of patients, meaning that SXBXP can significantly improve the quality of life of patients. CTFC is the most objective indicator of the disease which directly reflects the coronary blood flow, showing that SXBXP can significantly improve the blood flow of each branch of the coronary artery, and then increase myocardial blood perfusion and improve the symptoms of patients. Subgroup analysis and comparison of CTFC of the LAD, LCX, and RCA showed that SXBXP significantly improved coronary blood flow in the LAD (WMD = −6.36) and RCA (WMD = −6.72) than in the LCX (WMD = −5.73). From an anatomical point of view, since epicardial arteries are normal in patients with CSF and they do not pose any resistance to blood flow (conductive vessels), the cause of slow blood flow is mainly related to the “small vessels” (resistance vessels) of <400 μm, which are the most important parts regulating myocardial blood flow (Wang and Nie, 2011). Wang et al. found that SXBXP significantly increased coronary flow reserve and decreased microcirculatory resistance, and these indicators directly reflected the function of coronary microcirculation, which is closely related to coronary blood flow velocity (Wang et al., 2021). Basic studies have revealed that the active components of SXBXP, such as cinnamaldehyde and ginsenosides, produce pro-angiogenic effects by stimulating VEGF, PI3K/Akt/mTOR, and MAPK pathways, which are responsible for promoting angiogenesis, enhancing endothelial cell proliferation, mobilization, and tube formation, and increasing the number and density of microvessels (Lu et al., 2018; Hua et al., 2021). In addition, SXBXP can reduce the resistance to blood flow by upregulating cyclooxygenase two and downregulating intercellular adhesionmolecule-1, which dilates both large and small coronary vessels (Fang et al., 2017). These may be the intrinsic mechanism by which SXBXP increases coronary blood flow velocity, and the improvement of angina symptoms and the increase of exercise tolerance are the external manifestations of increased coronary blood flow velocity and increased blood perfusion.

ET-1 and NO, a pair of vasoactive substances with antagonistic effects, play an important role in maintaining the normal physiological function of blood vessels (He et al., 2018). ET-1 has a strong and persistent vasoconstrictive effect and plays a major role in small artery stiffening, endothelial dysfunction, and microcirculatory disorders (Smith et al., 2016; Coelho et al., 2018). In contrast, NO is a potent endogenous vasodilator and also has the effect of inhibiting platelet aggregation and vascular smooth muscle cell proliferation (Tettey et al., 2021). Studies have shown that impairment of endothelial function is closely related to the occurrence of CSF, and the degree of impairment of endothelial function is negatively correlated with the plasma NO level, but positively correlated with the ET-1 level (Ma et al., 2013; Jia et al., 2016). Reactive oxygen species and oxidative stress are the main pathological mechanisms of endothelial dysfunction (Ungvari et al., 2018). Experimental studies have shown that SXBXP can reduce H_2_O_2_-induced apoptosis in endothelial cells and increase cell proliferation activity (Lu et al., 2018). The basic mechanism may be that SXBXP increases the expression of superoxide dismutase and reduces the expression of malondialdehyde and NADPH oxidase, resulting in antioxidant effect (Ning et al., 2011). Inflammation is an important pathogenic factor causing many cardiovascular diseases, hs-CRP is the most important and most sensitive non-specific measure of reactive inflammation, and several studies have shown that the occurrence of CSF is specifically and significantly positively correlated with the hs-CRP level (Barutcu et al., 2007; Cetin et al., 2014). In addition, inflammation is strongly associated with endothelial dysfunction and has been identified as a possible “trigger” for structural and functional abnormalities in “small” coronary vessels in patients with CSF (Chalikias and Tziakas, 2021). Basic research has found that SXBXP reduces inflammatory responses by blocking the Ca^2+^/CaMKII/TLR/NF-kB axis (Tao et al., 2015). Numerous clinical studies have also confirmed the role of SXBXP in improving endothelial function and reducing inflammatory response (Wu et al., 2022). In this study, the ET-1 and hs-CRP levels were significantly reduced and the NO level was increased in the SXBXP group, suggesting that SXBXP may improve blood flow and symptoms in CSF patients by improving endothelial function and reducing inflammatory response.

## 5 Innovations and clinical implications

Clinical and scientific researchers have long paid relatively little attention to CSF, and its treatment options have progressed poorly so far. This study systematically evaluated the efficacy and safety of SXBXP in the treatment of CSF, which is the first of its kind in both western and Chinese medicine, providing relatively high-level evidence for the treatment of this disease and filling a gap in related studies. In addition, previous studies in TCM have focused mostly on changes in subjective indicators such as clinical symptoms and signs of patients, with insufficient attention to objective laboratory and clinical trials. This study focused on more objective clinical and laboratory indicators, including coronary angiographic findings, treadmill exercise tests, ET-1, NO, and hs-CRP, which are more readily accepted by a wide range of health care professionals. More importantly, SXBXP is used to treat a wide range of coronary diseases, and its role in non-obstructive coronary artery disease has been gaining attention in recent years. Previous study has demonstrated that it increases coronary flow reserve and reduces microcirculatory resistance, while the present study found that it significantly increases coronary flow velocity, improves endothelial function and reduces inflammatory responses, which greatly enriches its role in coronary small vessels.

SXBXP is recommended for angina pectoris due to CSF in the Chinese Expert Consensus on SXBXP for Angina Pectoris published in 2018 (Fan et al., 2018). The latest 2022 version of the consensus adopted more stringent evidence review rules, which resulted in the removal of CSF indications due to the lack of a systematic summary of clinical evidence on the treatment of CSF with SXBXP. GRADE is a common method for rating the quality of clinical evidence and is widely used in the preparation and revision of guidelines and expert consensus. This study provides a GRADE rating for the quality of evidence based on meta-analysis, which will strongly contribute to the revision of the relevant consensus. The rigor of the clinical study design directly affects the reliability of the findings, and we recommend a rigorously designed multicenter, placebo-controlled, large-sample RCT in compliance with the CONSORT statement to further validate its efficacy and safety (Calvert et al., 2013).

## 6 Limitations

This study has the following limitations: Only two of the 10 studies described the specific randomization method properly, while the rest only mentioned randomization and did not specifically describe the randomization method, and all did not mention the use of allocation concealment and blinding method, thus potentially creating a risk of bias in the study. The efficacy measures lacked the endpoint event measures such as the incidence rate of cardiovascular events, and there were few data on safety. The occurrence of adverse reactions was reported only in two studies. The use of CWM was not the same in all studies, and the span of treatment courses was large in different studies, which may lead to an increase in clinical heterogeneity. GRADE evidence quality ratings are mostly low or very low, and relevant results should be treated with caution. Therefore, more multi-center, large-sample, randomized, double-blind placebo studies with rigorous design and reasonable protocols are needed to verify the efficacy and safety of SXBXP in the treatment of CSF.

## 7 Conclusion

Although some results in this study suggest that there may be a positive effect of SXBXP for CSF, the quality of the primary study including the reporting is too poor and therefore, no benefits could be demonstrated. High-quality RCTs with more samples, scientific design, multiple centers and strict implementation are still needed to further investigate the efficacy and safety.

## Data Availability

The original contributions presented in the study are included in the article/Supplementary Material, further inquiries can be directed to the corresponding author.
