# Supplementary material for: Meta-analysis and trial sequential analysis of shexiang baoxin pill for coronary slow flow
Source: Front Pharmacol. 2022 Aug 22;13:955146. doi: 10.3389/fphar.2022.955146 (PMC9441803; doi:10.3389/fphar.2022.955146)
Supplement: Supplementary file 1 [file Table1.DOCX]

| **Supplementary material S1** Source, compositions, extraction procedure, actions, indications, administrations and chemical analysis of Shexiang Baoxin Pill (SXBXP) | | | | | | | | |
| --- | --- | --- | --- | --- | --- | --- | --- | --- |
| **Source** | **compositions** | **Processing of each composition** | **Extraction procedure** | **Actions** | **Indications** | **Administration and dosage** | **Quality control reported? (Y/N)** | **Chemical analysis  reported? (Y/N)** |
| Shanghai Hehuang  Pharmaceutical Co., Ltd. | *Moschus berezovskii* Flerov., *Moschus sifanicus* Przewalski or *Moschus moschiferus* Linnaeu. [Cervidae, Moschus Artifactus] | Remove the sac wall of “Maokeshexiang”, take out the “Shexiangren” and eliminate foreign matter'. Pulverize before use. | Pulverize the six ingredients except Styrax to fine powder, make pills with Styrax and a quantity of white wine and dry. | To warm and unblock meridians with aromatic medicinals, replenish qi and strengthen heart. | Chest *bi* disorder due to qi stagnation and blood stasis, manifested as fixed pain in precardium; Angina pectoris and myocardial infarction due to myocardial ischemia with the symptoms described above. | 22. 5 mg per pill.  For oral administration, 1-2 pills per time, three times a day. | Y -  ZYB20794071 issued by National Medical Preducts Administration  [Detail information can be got from https://www.nmpa.gov.cn/xxgk/ggtg/zhybhpzh/zhybhpzhgg/19940206010101501.html] | Y -  HPLC  [Detail information can be got from Pharmacopoeia of the People's Republic of China (Part I, finished preparations and single flavor preparations, Shexiang Baoxin Wan)] |
|  | *Panax ginseng* C. A. Mey. [Araliaceae, Ginseng Radix et Rhizoma] | Soften thoroughly, cut into thin slices, and dry, or pulverize or break to pieces before use. |  |  |  |  |  |  |
|  | *Bos taurus domesticus* Gmelin. [Bovine, Bovis Calculus Artifactus] | Artificial Cow-bezoan is prepared with powder of cow bile, cholic acid, hyodeoxycholic acid, taurine, bilirubin, cholesterol and trace elements, etc. |  |  |  |  |  |  |
|  | *Cinnamomum cassia* Presl. [Lauraceae, Cinnamomi Cortex] | Eliminate foreign matter and rough bark. Pound to pieces before use. |  |  |  |  |  |  |
|  | *Liquidambar orientalis* Mill. [Hamamelidaceae, Styrax] | Purify Styrax to get purified balsam |  |  |  |  |  |  |
|  | *Bufo bufo gargarizans* Cantor *or Bufo melanostictus* Schneider (Bufonidae, Bufonis Venenum) | Break Bufonis Venenum to pieces, macerate with white rice wine, frequently stir until become concentrated extract, dry and pulverize. |  |  |  |  |  |  |
|  | *Cinnamomum camphora* (L.) Presl [Lauraceae, Borneolum] | Steam distillation. |  |  |  |  |  |  |

**Reference:**

Committee of National Pharmacopoeia. (2015). *Pharmacopoeia of the People's Republic of China*. Chemical Industry Press.
