# Supplementary material for: Meta-analysis and trial sequential analysis of shexiang baoxin pill for coronary slow flow
Source: Front Pharmacol. 2022 Aug 22;13:955146. doi: 10.3389/fphar.2022.955146 (PMC9441803; doi:10.3389/fphar.2022.955146)
Supplement: Supplementary file 2 [file Table8.DOCX]

**Supplementary material S8** subgroup analysis of corrected TIMI frame count in right coronary artery (CTFC-RCA) based on treatment duration, average age, gender distribution, and sample size

subgroup analysis of CTFC- RCA based on treatment duration

subgroup analysis of CTFC- RCA based on average age

subgroup analysis of CTFC- RCA based on gender distribution

subgroup analysis of CTFC- RCA based on sample size
