# Supplementary material for: Meta-analysis and trial sequential analysis of shexiang baoxin pill for coronary slow flow
Source: Front Pharmacol. 2022 Aug 22;13:955146. doi: 10.3389/fphar.2022.955146 (PMC9441803; doi:10.3389/fphar.2022.955146)
Supplement: Supplementary file 4 [file Table9.DOCX]

**Supplementary material S9** subgroup analysis of endothelin-1 (ET-1) based on treatment duration, average age, and gender distribution

subgroup analysis of ET-1 based on treatment duration

subgroup analysis of ET-1 based on average age

subgroup analysis of ET-1 based on gender distribution
