# Supplementary figures and images for: Meta-analysis and trial sequential analysis of shexiang baoxin pill for coronary slow flow
Source: Front Pharmacol. 2022 Aug 22;13:955146. doi: 10.3389/fphar.2022.955146 (PMC9441803; doi:10.3389/fphar.2022.955146)

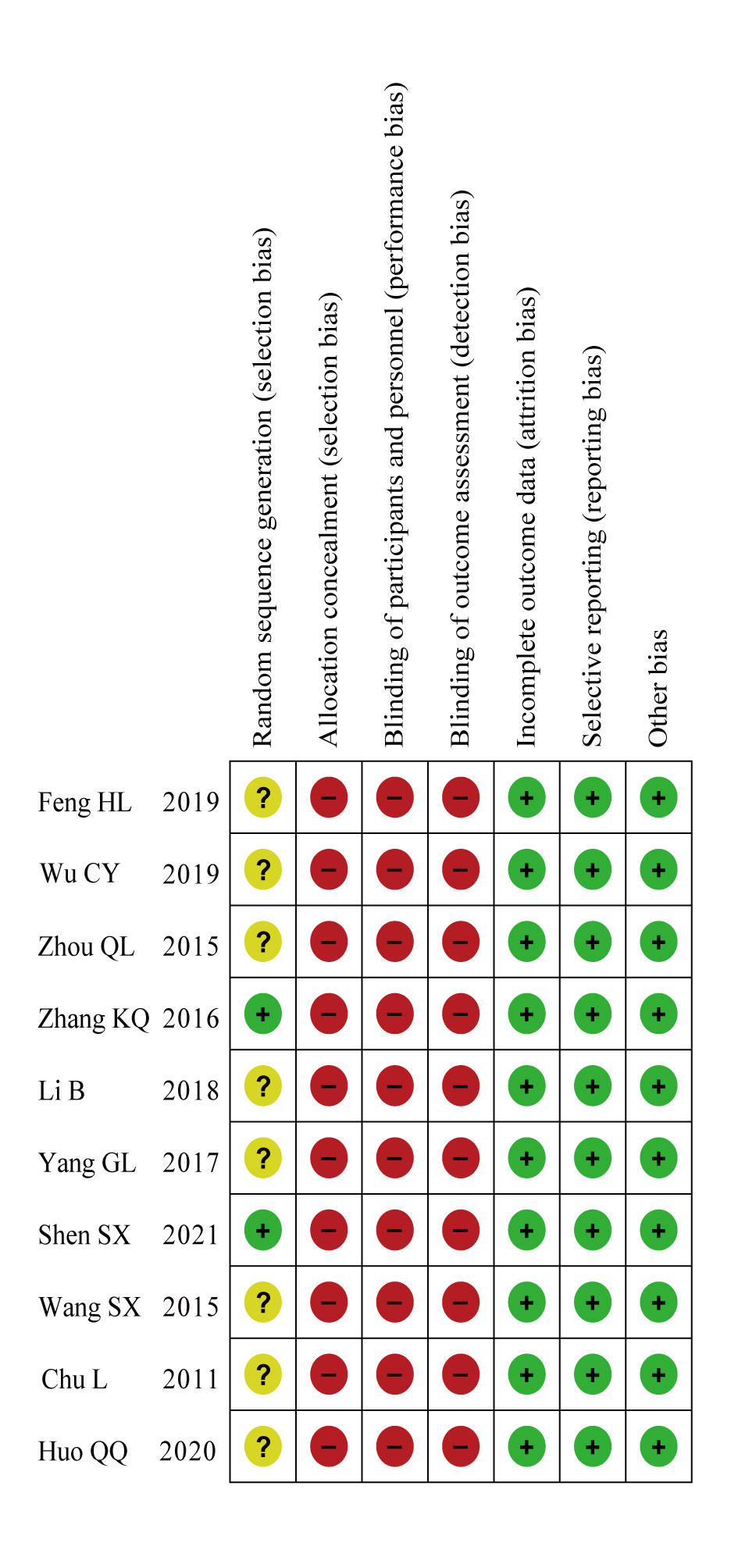


Supplementary material S5 Risk of bias summary graph

Supplement: Supplementary file 6 [file Table5.DOCX]

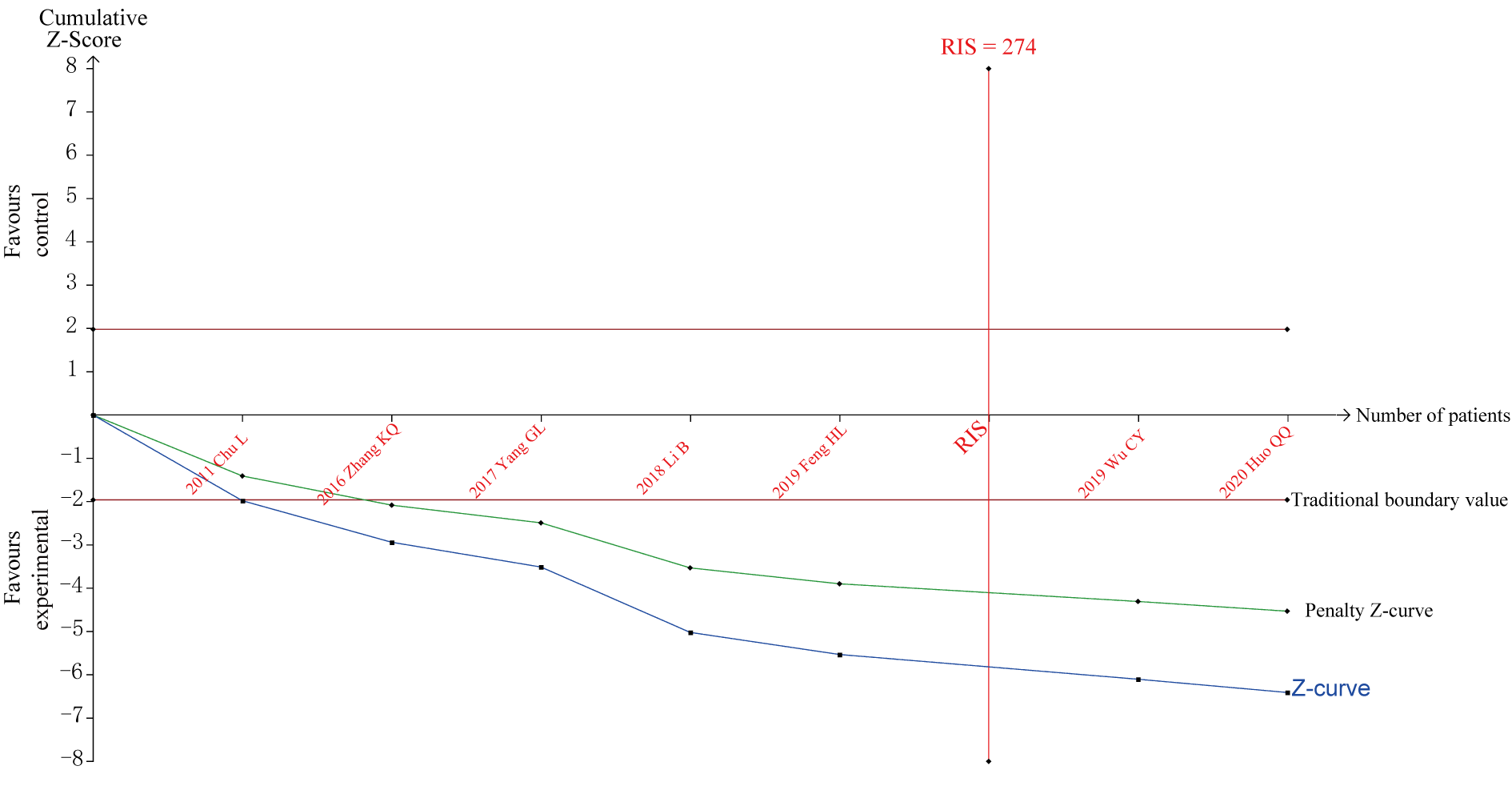


Supplementary material S11 Penalty analysis of efficacy on angina pectoris

Supplement: Supplementary file 9 [file Table11.DOCX]
