# Supplementary material for: Meta-analysis and trial sequential analysis of shexiang baoxin pill for coronary slow flow
Source: Front Pharmacol. 2022 Aug 22;13:955146. doi: 10.3389/fphar.2022.955146 (PMC9441803; doi:10.3389/fphar.2022.955146)
Supplement: Supplementary file 7 [file Table3.DOCX]

| CSF | Coronary slow flow |
| --- | --- |
| SXBXP | Shexiang Baoxin Pill |
| TCM | Traditional Chinese medicine |
| RCT | Randomized controlled trial |
| TSA | Trial sequential analysis |
| GRADE | Grades of Recommendation, Assessment, Development and Evaluation |
| CWM | Conventional western medicine |
| RR | Relative risk |
| WMD | Weighted mean difference |
| CI | Confidence interval |
| NO | Nitric oxide |
| CTFC | Corrected TIMI frame count |
| LAD | left anterior descending artery |
| LCX | left circumflex artery |
| RCA | right coronary artery |
| ET-1 | endothelin-1 |
| hs-CRP | high-sensitivity C-reactive protein |
| CNKI | China National Knowledge Infrastructure |

**Supplementary material S3** the abbreviations used in this manuscrip
