# Supplementary material for: Meta-analysis and trial sequential analysis of shexiang baoxin pill for coronary slow flow
Source: Front Pharmacol. 2022 Aug 22;13:955146. doi: 10.3389/fphar.2022.955146 (PMC9441803; doi:10.3389/fphar.2022.955146)
Supplement: Supplementary file 8 [file Table4.DOCX]

**Supplementary material S4**

**PubMed search strategy**

**#1** "Coronary Vessels"[MeSH Terms] OR "coronary artery"[Title/Abstract] OR "coronary"[Title/Abstract]

**#2** "slow flow"[Title/Abstract] OR " slow blood flow"[Title/Abstract] OR "slow flow phenomenon"[Title/Abstract]

**#3** #1 AND #2

**#4** "Shexiang Baoxin Pill"[Title/Abstract] OR "Shexiang Baoxin"[Title/Abstract]

**#5** ("randomized controlled trial"[Publication Type] OR "controlled clinical trial"[Publication Type] OR "randomized"[Title/Abstract] OR "placebo"[Title/Abstract] OR "clinical trials as topic"[MeSH Terms:noexp] OR "randomly"[Title/Abstract] OR "trial"[Title]) NOT ("animals"[MeSH Terms] NOT ("humans"[MeSH Terms] AND "animals"[MeSH Terms]))

**#6** #3 AND #4 AND #5
