# Supplementary material for: Meta-analysis and trial sequential analysis of shexiang baoxin pill for coronary slow flow
Source: Front Pharmacol. 2022 Aug 22;13:955146. doi: 10.3389/fphar.2022.955146 (PMC9441803; doi:10.3389/fphar.2022.955146)
Supplement: Supplementary file 10 [file Table6.DOCX]

**Supplementary material S6** subgroup analysis of corrected TIMI frame count in left anterior descending artery (CTFC-LAD) based on treatment duration, average age, gender distribution, and sample size

subgroup analysis of CTFC-LAD based on treatment duration

subgroup analysis of CTFC-LAD based on average age

subgroup analysis of CTFC-LAD based on gender distribution

subgroup analysis of CTFC-LAD based on sample size
