# Supplementary material for: Meta-analysis and trial sequential analysis of shexiang baoxin pill for coronary slow flow
Source: Front Pharmacol. 2022 Aug 22;13:955146. doi: 10.3389/fphar.2022.955146 (PMC9441803; doi:10.3389/fphar.2022.955146)
Supplement: Supplementary file 11 [file Table10.DOCX]

**Supplementary material S10** subgroup analysis of nitric oxide (NO) based on gender distribution

subgroup analysis of NO based on gender distribution
